# Pulmonary Consumption Cured by Persistent Walking, and without Medicine

**Published:** 1912-05

**Authors:** 


### TEXAS MEDICAL JOURNAL.

Established July, 1885

F. E. DANIEL, M. D., - - - - - Editor, Publisher and Proprietor

Published Monthly.—Subscription, $1.00 a Year.

VOL. XXVII. AUSTIN, MAY, 1912. No. 11.

The publisher is not responsible for the views of the contributors.

### Original Articles.

For Texas Medical Journal.

### Pulmonary Consumption Cured by Persistent Walking, and Without Medicine.

(BY A TEXAS PHYSICIAN LONG RETIRED FROM PRACTICE.)

CASE NO. 1.—A lady of about 30 years of age came to visit and consult me. It was the first case to whom I suggested the idea of cure by the exercise of walking, so far as I can remember. She was my sister-in-law, and I urged her to let me try the experiment on her. She thought, at first, that a cure by exercise alone, and without medicine, was nearly in the nature of a joke, but finally consented. As she was very feeble, I began cautiously. A careful examination indicated that she was well along in the second stage, perhaps, of consumption. At first, she thought that she could not walk at all, except across the room. I got her to walk about one hundred feet, after breakfast, by my assistance; then we repeated in the middle of the day, and near nightfall. This distance three times a day, continued three days, then increased to two hundred feet during four days. On the eighth, she began to walk, unaided, and increased distance to about three hundred feet. On the fifteenth, by gradually increasing, she went eight or nine hundred feet, and by the twentieth she was able to increase the distance to about eighteen hundred; then she increased rapidly until the end of the sixth week, at which time four miles per day was an easy matter. As she was then relieved of every symptom, she went home, feeling well again. This seems incredible, yet I do not exaggerate. It was a remarkable cure. The pathological condition of the lungs might not, possibly, have been so severe as the symptoms and physical signs indicated.

In taking the walking exercise, the distance, of course, need not be accurately measured, the idea being to gradually increase for awhile; then, as the patient shows improvement, increase more rapidly, and then hold to the fixed, unalterable idea that the exercise must be persistently continued day after day, rain or shine, except when the fever runs high, then leave off until it is lowered considerably. It should be remembered that the exercise of walking is equable, and never violent, gradually increasing the volume of circulation to a normal standard, thereby secondarily promoting a desire for food, and the ability to digest it. It also gradually increases the number, conservatively, of the respiratory functions, affording more oxygen for the overtaxed lungs. Bad weather obstructions are overcome by the use of a raincoat, overalls, and an umbrella, in addition to good and thick flannel underwear.

Case No. 2.—In addition to the walking exercise, I put an oil silk jacket on a negro washerwoman, who had severe hemorrhage occasionally. After two weeks, she had no more hemorrhages, and was completely restored to health.

Case No. 3 was a nephew of mine from North Texas. He was about 21 or 22 years old, and delicately constituted. His mother was strong and healthy, but his father had died with consumption. When he came to me, he had a wretched cough, night sweats, great debility, and was greatly emaciated. I put him to walking gradually, and managed him just as I did case No. 1. Like all other consumptives, he disliked exercise, and it was by great firmness and insistence that I kept him going for awhile; then he braced up, and at the end of two months was well again, and walked four miles a day without fatigue. I bought him a rain suit, overalls and umbrella, to walk, rain or shine.

Case No. 4.—A gentleman from Hillsboro was in a bad way with his lungs, and had been advised to go to Monterey, Mexico. It was winter time with us at Austin. He came to see me, and I advised him to try the walking plan while there. He took readily to the idea, and informed me afterwards that he commenced the next day after his arrival, and improved from the jump. He got well so fast that at the end of thirty days he felt so well that he left for home in fine condition. He walked persistently three times a day. His condition might not have been so far advanced as others whom I mention.

Case No. 5.—A young lady of about 20. A decidedly welldeveloped case of tuberculosis, with occasional violent hemorrhage. This lady was quite emaciated; had come from the North; know nothing of family history. She promised to do just as I suggested in all things, if I would advise her. She boarded at a hotel where I did not see her often. She, too, was astonished when I said that she must walk, and that no medicine was needed. She was afraid that exercise would increase the tendency to hemorrhage. "No," said I, "you will bleed, anyway, and without hope, if you take no exercise." She had faith somehow, and followed my instructions, without my help, and to the letter. In about four months she came to see me at the institution, much to my surprise, for I had forgotten her. As she was so young, and as consumptives are so much averse to exercise, I thought she would never attempt to carry out my instructions, but she did. She said she was twenty pounds heavier, and had had only one hemorrhage, which occurred the first week; had a fine appetite; no cough whatever, and slept well. She came to say that she was pleased with Austin, had become acquainted with many University students, and wanted to know if it would hurt her to enter and finish up some of her unfinished home studies. I said, "Yes, if you agree to walk from your hotel to the University, taking regular exercise every day. You ought to continue exercise for one year at least." I saw her once or twice after that day, but somehow I have a dim recollection that she had to return for awhile to her home in the North, but went away intending to come back, as I had advised her to remain in this climate.

Case No. 6.—A Northern man—consumptive. He had tried San Antonio, and had got no benefit, but an acquaintance in Austin had induced him to try that city. He had been in town about one month, and felt a little better. The acquaintance brought him to me for advice. He promised, and did walk with advantage until his cough and strength were much improved. He felt like he could do something to help pay his board. I sent him, with recommendation, to Radam, the gardener, advising him to drive a vegetable wagon, for it would shake him up, and keep up the exercise. I told him to always take a blanket along, so that if he became overfatigued he could stop and lie down for a thirty-minute rest, then go on, and go rain or shine, in his raincoat, hat and overalls. He got the job, and religiously observed my advice. Six months after I met him at the postoffice, and he was delighted to meet me, but mortified to find that I did not know him. "Well, how can I know you when I never saw you before." "And," said he, "you don't know the consumptive you made drive a vegetable wagon for Radam?" He was so fleshy I could not recognize him, and then he had, after so long a time, slipped out of my memory. Two years after this meeting, I met him near the same place, and he was, as before, profuse in his expressions of delight, but, alas! I knew him not, and he was puzzled, and regretted my forgetfulness; but how could I help it; he weighed, so he said, over 225 pounds. Said he, "I am now going to do another thing you advised. You told me to go into the cattle or sheep business, and then 'cut the bridge behind me,' that I may never go North again. I have money in my hands to send to my only relatives—my mother and my sister—who have promised to come and live with me." I have never heard from him since.

CASE NO. 7.—A case of singular cure. A farmer who had been regarded for some time as a sure victim of consumption, having all of the recognized orthodox signs and symptoms. He complained to me that he often caught fresh cold, and it always aggravated his condition. I thought over the matter, and merely suggested something, as I intended to prevent the tendency to colds. I told him to get a deep tin bucket that would be deep enough to hold both feet and allow the water to reach up to his knees. He must fill it with water, of the temperature of ordinary well water (surface well) and put his feet in, and hold them there about ten minutes at a time, for three or four different days, then twenty minutes each bath ever afterwards. This bath to be taken regularly every morning as soon as he arose. From some cause, which I do not remember, I never saw him again until he came into my office, as he said, to meet the insurance doctor, who would examine him for life insurance. I thought he had either died or had moved away. "How can you, Dick," said I, "offer for insurance when your lungs are so diseased?" "Shucks," said he, "didn't you cure me by making me keep my feet clean? That business broke up my consumption completely, in a year, and I am as sound as a dollar." The visiting physician gave him insurance, and remarked that the man evidently had had many cavities in his lungs, at one time, but all were healed, and he was suitable for insurance. The patient lived many years longer, and died with some acute disease which did not affect his lungs. He said that I was a wonderful doctor. Strange to say, from some unaccountable reason I never did try the experiment on any other patient, in that way. His cough began to improve, he said, not long after he had begun to take the baths. In the foregoing cases, it might have been, if I had thought of it, a happy combination to have the patients to take foot baths as well as the walks. This plan would not interfere with medical treatment of any kind, if a physician desired to prescribe drugs for internal use. Exercise brings desire for food and the ability to digest, and the bath lessens tendency to fresh colds and invigorates the system. Medicine and continued rest, I am afraid, debilitates the patient and never stimulates the desire for food. Exercise, however, in the last stages of consumption, will rarely, if ever, do any good, but the foot baths might alleviate the distressing cough to some extent. If necessary, the baths could be tepid, or nearly so, at the beginning. Milk curdled by Fairchild's essence of pepsin, as directed on the bottle, in liberal quantity, with soft boiled eggs, makes an ideal diet for consumptives,—adding good tender beef after awhile.

CASES NOS. 8 AND 9.—These two are beautiful cases to illustrate the benefit of walking—by comparison. One followed my advice; the other did not. Both were under my care in an institution, and both became afflicted with symptoms of consumption about the same time, and were about the same age, one being slightly heavier than the other. Both were nearly of the same temperament. After a year or so, I took more notice of them, for the nurse called attention to the increased debility and occasional fever. I explained to them my opinion about the benefit of regular exercise by persistent walking, and urged them to try it, for they were yet able to walk about, to some extent. One of them caught the idea and the spirit, and finally acquired the habit of regular exercise. She was able to stay her time out in school; got well, and is at home in fine health. The other could not have courage to hold up regularly; got dispirited, and was buried a few years ago, a sad victim of consumption. I have sometimes since blamed myself for not requiring her to walk, for she had the same opportunity to try for life that the other girl had, and perhaps needed pushing only.*

### TREATMENT OF MALARIAL FEVER WITH COLD WATER, WATERMELONS AND SOME CALOMEL.—IN THE SUMMERTIME, 1859 AND 1860.

This method (hereinafter described) of treating intermittent, bilious remittent and some forms of congestive fever was original, so far as I know, with a certain noted East Texas physician and planter, who, by the way, was my preceptor. The practice then

*NOTE.—The patient who died was tried with *fresh air* treatment, by allowing her to sleep on an open upper gallery—south side of building—for a long time, but somehow she did not improve as was expected. Really she needed exercise to create a desire for food and promote digestion.

was to avoid the use of cold water for fear of giving the patient a severe cold, or possibly induce pneumonia or some other complication. The old doctor's idea was to reduce the fever as quickly as possible, thereby preserving the strength, and possibly prevent continued fever. It was, as a remedy, most grateful to the patient—the men sometimes swearing that it was glorious; and, then, there was no real danger in it. I have seen the doctor, when his patient was burning up with fever, pulse full and bounding, tongue furred, racking headache, and vomiting bile every few minutes, take a bucketful of cool well water (it's cold enough) and a cocoanut dipper, strip his patient naked, lay him on a sheet spread upon the floor, then pour the water gradually all over him or her, beginning at the head and going on down even over the lower extremities, continuing until the "goose bumps," as he called them, would appear, or until the patient felt somewhat cool. Then he would wrap him up in bed with quilts or blankets. Usually in twenty minutes the patient would drop off to sleep, and sweat his fever off in from one to two or three hours. If, however, the nausea was not entirely subdued by the water, a watermelon was cut, and the patient, to his delight, was allowed to eat, solidly, the half or the whole of the heart of a ripe watermelon, which always quieted him and allowed him to sleep. The heart, or even any part, of a watermelon acts as a fine adjuvant, for as an allayer of severe nausea, an eliminative and a diuretic there is nothing, in my opinion, to equal it.

Case No. 1.—I inherited this method of treatment from the doctor, and it so happened, soon after the Civil War, that a former Confederate soldier, of the town where I practiced, found a comrade of his who was his lieutenant in Hood's famous Texas brigade, lying sick with fever in Polk county, about fifty miles away. He implored "Billy" to get him to a doctor who would allow him some cold water, for he was tired of hot water, and was burning up. "I can do that very thing," said Billy. He got a carryall, so called in those days, put a mattress in it, and after traveling all night, brought him to me at my hotel. "Here is the man," said Billy, "who will give you cold water and some watermelon." The man was somewhat flighty occasionally, and was in a great nervous tremor. I made the necessary preparations and then poured cool well water all over him. As he began to revive he cried out, as he caught at the stream with his mouth: "My God, doctor, that is glorious, may you live forever." It was really an affecting scene, and I felt good, for I knew that I was using the treatment which would give him the quickest relief, and I was justified, for in not less than twenty minutes after his eating the melon, and being wrapped up, he was asleep, and soon sweated profusely. In two or three hours he awoke free from fever, and "feeling fine," he said. He made a good recovery, and was my fast friend forever after.

Case No. 2.—I visited a member of the Legislature in my town, a close friend of mine, who, while I was absent, was attacked with bilious remittent fever. I was called as soon as I returned, and found that his fever had continued without abatement for about four days. The primae viae had been cleaned out by several doses of calomel. As in the Polk county case, he was much debilitated, had nausea and nervous tremors. After the cool water bath, during which he, too, caught at the water, I allowed him some heart of a watermelon, which had been lying in a tub of cool well water. The result was just about the same as in the other case, and he became a firm convert to the treatment of fever by cool water. I say "cool" because I doubt the advisability of using ice cold water in cases like these, for you can not use the water so long and so freely without some real risk of producing an undue chilliness. The physician, who, once upon a time, went to New Orleans to treat yellow fever with cold water, had ice rubbed over the body, but that was his great mistake, for when he had it rubbed over himself, when down with the fever, he as well as his selected patients died, and died early. If he had used, with judgment, my preceptor's plan, he and many of his patients might have fared better.

Case No. 3.—A lady—my wife—lay low with fever and excessive nausea. My old preceptor, her father, being called, we decided to stop the nausea with watermelon, for temporary relief at least. With heartfelt gratitude we found it was the very thing to do, for it acted "like a charm," indeed, stopping the nausea at once, and to our astonishment relieved all dangerous symptoms without bathing with cool water and without further treatment except tincture of iron, to provide rapid convalescence. This may appear marvelous to those who have never tried this remedy for nausea, as it occurs in fever patients. The question arises, how does watermelon do such effective work in these cases? I pass this question up to you, that you may enlighten me. Is it the mere effect of weight and its coloring effect upon the mucous membrane of the stomach? Take the case.

The cases previously mentioned are only a few of the many similar cases treated by us with water and melons. In our practice it was no uncommon thing to have like conditions confront us every week, and for us to treat them as we did the patients mentioned, hence our experiences were not confined to the limited number of three cases. I have no remembrance of bad effects from the use of cool water, for it was not used indiscriminately, regardless of patient's strength, or complications of any kind.

